# Supplementary material for: Yiai Fuzheng decoction inhibits triple-negative breast cancer by remodeling the immune microenvironment
Source: Front Immunol. 2025 Sep 30;16:1615631. doi: 10.3389/fimmu.2025.1615631 (PMC12518410; doi:10.3389/fimmu.2025.1615631)
Supplement: Supplementary file 2 [file DataSheet1.zip › Supplementary File 2/Treatment/C_vs_D/07_Pathway_Analysis/Enrichment_Analysis/smpdb_pathway/Network.pdf]

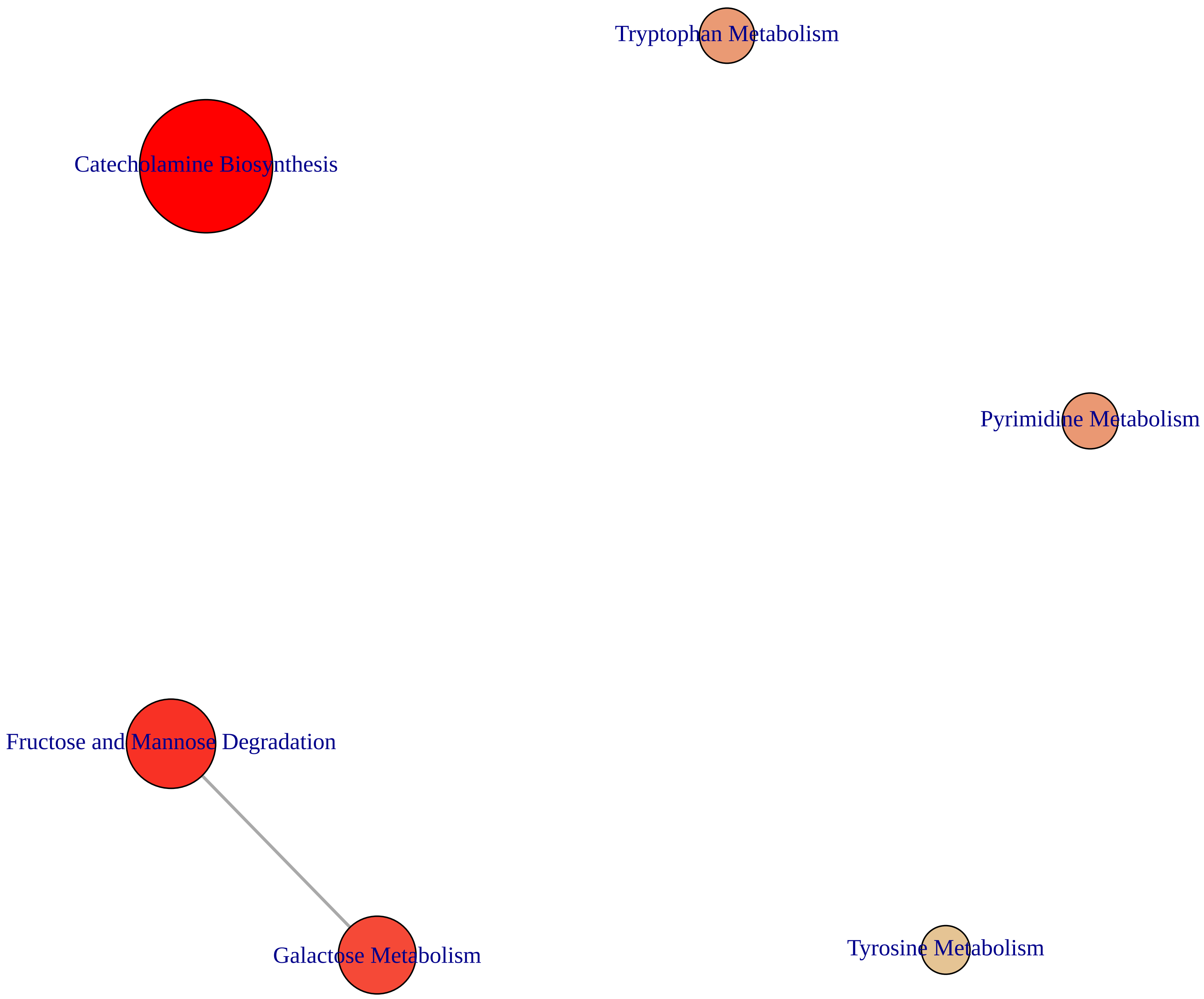
Tryptophan Metabolism
Catecholamine Biosynthesis
Pyrimidine Metabolism
Fructose and Mannose Degradation
Galactose Metabolism
Tyrosine Metabolism
